# Practical Application of the Five Domains Animal Welfare Framework for Supply Food Animal Chain Managers

**DOI:** 10.3390/ani12202831

**Published:** 2022-10-18

**Authors:** Temple Grandin

**Affiliations:** Department of Animal Science, Colorado State University, Fort Collins, CO 80526, USA; cheryl.miller@colostate.edu; Tel.: +1-970-443-1510

**Keywords:** animal welfare, auditing, supply chain, Five Domains

## Abstract

**Simple Summary:**

The Five Domains model is being increasingly used as a framework for assessing animal welfare on farms. This commentary is focused on the practical application of the Five Domains by supply chain managers who buy food animal products and often work in global supply chains. Assessments used in commercial supply chains need to be simpler than assessment tools used in scientific research. There needs to be very clear guidance on conditions that should result in a failed audit. Welfare auditors can be easily trained to assess animal-based outcome measures such as body condition score, foot pad lesions on poultry or lameness. A farm would also have to have the type of housing that is specified in the buyer’s welfare guidelines. Easy to evaluate animal welfare indicators should be included in each of the four domains of nutrition, environment, health, and behavioral interaction.

**Abstract:**

The author has worked as a consultant with global commercial supply managers for over 20 years. The focus of this commentary will be practical application of The Five Domains Model in commercial systems. Commercial buyers of meat need simple easy-to-use guidelines. They have to use auditors that can be trained in a workshop that lasts for only a few days. Auditing of slaughter plants by major buyers has resulted in great improvements. Supply chain managers need clear guidance on conditions that would result in a failed audit. Animal based outcome measures that can be easily assessed should be emphasized in commercial systems. Some examples of these key animal welfare indicators are: percentage of animals stunned effectively with a single application of the stunner, percentage of lame animals, foot pad lesions on poultry, and body condition scoring. A farm that supplies a buyer must also comply with housing specifications. The farm either has the specified housing or does not have it. It will be removed from the approved supplier list if housing does not comply. These types of easy to assess indicators can be easily evaluated within the four domains of nutrition, environment, health and behavioral interactions. The Five Domains Framework can also be used in a program for continuous improvement of animal welfare.

## 1. Introduction

Both animal welfare researchers and commercial buyers of animal products are moving towards adoption of the Five Domains Model for their animal welfare programs [1,2,3]. The author has worked extensively as a consultant with commercial supply managers on both training of animal welfare auditors and development of their auditing programs [4,5]. Most older animal welfare assessments, such as the Five Freedoms, emphasized the importance of preventing suffering [6]. The Five Domains model states that preventing suffering is not sufficient [1]. The animals must also have opportunities for positive emotional (affective) experiences. Research clearly shows that animals have affective experiences that are both positive and negative [7,8]. In the Five Domains model, there are four domains where animal welfare indicators can be assessed. They are: Nutrition, Environment, Health and Behavioral Interactions [2]. These four domains have either a positive or negative effect on the fifth affective domain that cannot be directly measured. The four domains are similar to the four parts of the European Welfare Quality Protocols of Good Feeding, Good Housing, Good Health, and Appropriate Behavior [9]. It was developed by European welfare specialists. This similarity will make it easier for supply chain managers to incorporate the Five Domains if they are already familiar with Welfare Quality. The first three domains are very similar. The main difference is in the fourth domain of Behavioral Interactions. Welfare Quality includes positive emotional states in the fourth principle. In the Five Domains, positive emotions are removed from this section and they become part of the fifth domain. It contains both positive and negative affective emotional states that cannot be directly measured. Before discussing specific ways to incorporate the Five Domains, the author is first going to discuss how commercial supply chains operate.

## 2. Commercial Supply Chain Managers Need Simple Easy-To-Use Guidelines

During the author’s many years of consulting work with many commercial buyers, supply managers, and producers, she has learned that if an assessment guideline becomes too complicated, they cannot effectively implement them. A welfare audit conducted by either a commercial auditing firm or a corporate buyer has to be able to be conducted in a single day on each farm. Assessments and audits that are used commercially must be simpler than measurements and assessments used in research. The commercial reality is, that a typical welfare auditor is trained in a two or three day workshop [10]. At the end of the training, they have to take an exam. To become fully qualified, they also have to conduct two or three shadow audits with an experienced auditor. There is also a requirement to attend either online or in person livestock meetings to fulfill requirements for continuing education. This is a very short period of training, compared to the studying that is required to become a veterinarian, or scientific researcher. Many corporations also have advisory boards or welfare officers that have advanced degrees. These people provide advice on a corporation’s animal welfare standards. The author has observed that an effective supplier auditing program for animal welfare has three components [11]. They are: (1) independent third party audits conducted by an auditing company, (2) Audits by corporate supply chain buyers, and (3) Internal self-audits by the farmer or slaughter plant [11]. Further observations by the author indicate that all three of these components are essential to help ensure that animal welfare guidelines are being followed.

A commercial supply chain manager also needs clearly written guidance on both poor practices and housing that would result in a failed audit. This is essential from a legal standpoint. When a supplier has to be “delisted” and removed from a company’s approved supplier list, the reason for failure has to be clear. The author observed a bad situation where a company that was delisted, sued a third party independent auditing company. The delisted supplier argued that the guideline was vague and did not clearly specify the reason for being delisted.

Wording in a guideline must not be vague [11]. For example, wording such as provide sufficient space or handle animals calmly is too vague. There is no easy objective way to train an auditor to assess this. Two examples of clear guidelines, are either a numerical space requirement for a housing, or a statement that all the animals must have sufficient space to all be able to lie down at the same time without resting on top of another animal. Photographs that show a correctly stocked pen and an overstocked pen are another easy method to provide guidance [12]. Another example of vague unclear guidance is the term “unnecessary suffering”. This statement appears in both the United Kingdom and Irish animal welfare acts [13,14]. This will not be effective in a commercial system because there are too many different ways that it can be interpreted.

### 2.1. Non-Compliances That Would Result in an Automatic Failed Welfare Audit

Many existing welfare guidance documents that are used commercially or by a government have criteria for severe animal welfare problems. Most of these conditions or abusive acts by people would result in being delisted in a commercial system or major regulatory penalties in a government system. From a supply chain manager’s viewpoint, it is essential that the wording is very clear.

### 2.2. Acts of Abuse or Neglect–Automatic Failure

Managers of supply chains need to enforce severe penalties on suppliers who allow acts of abuse to occur. Photos of people abusing animals or neglected health problems can go viral online. This may be really costly for a food company.

Below is a list of Acts of Abuse that would result in an automatic failed audit. 

During a slaughter audit, cutting or dismembering an animal that is showing signs of returning to consciousness [15,16].

Allowing conscious poultry or pigs to enter the scaulder.

Acts of abuse during handling such as dragging conscious non-ambulatory animals [16] beating animals [15,16,17], breaking tails [17,18] Poking sensitive areas of the animal such as rectum, eyes, mouth, ears, or udder [15], or lifting sheep or goats by the wool or horns [19].

Severe neglected health problems such as necrotic prolapses, necrotic ocular neoplasia that has invaded the face or deep infected cuts.

### 2.3. Key Welfare Indicators

Key welfare indicators are animal-based measurements that seriously compromise animal welfare. These indicators are scored and tabulated as the percentage of the animals that have a condition that is a serious welfare problem. Other names for these indicators are core criteria [15] critical control points or critical non-compliances. They identify most important welfare problems. One example that has been determined by experts is lameness (difficulty walking) [20]. In the broiler poultry industry, the author is currently working with a buyer who is in the process of adopting three major key welfare indicators for broiler chickens. Two of them are foot pad lesions and hock burn and they will be used in a global supply chain. These two indicators are associated with poor housing and they can be easily measured at slaughter. The original Welfare Quality protocols were too time consuming for use in many commercial systems. Shorter versions that use the key indicator concept are being developed for indoor beef cattle and have already been implemented for dairy cattle [20,21]. 

### 2.4. Animal Housing Specifications

If a commercial guideline prohibits certain types of housing such as sow gestation stalls or small battery cages for laying hens, the audit is failed if the prohibited housing is being used. The guideline should clearly state the types of housing that are definitely not permitted. The housing guidelines must also clearly state things that are required. Some examples may be pasture access for dairy cows, nest boxes for laying hens or straw bedding for pigs. Both the supply chain manager and the auditor need clear guidance on both prohibited forms of housing and required housing features. The guideline should not be too prescriptive on permitted housing, because producers need to be free to innovate.

### 2.5. Problems with a Single Combined Welfare Score

The Welfare Quality system [9] attempted to convert a large number of welfare measurements into a single score. One of the problems with a single score is that a serious welfare problem may be concealed [22]. In one study, this enabled a dairy with 47% lame cows to pass a welfare audit because they had high scores on other welfare indicators such as access to clean water [23]. Other researchers have also found problems with converting animal welfare data into a single score [24]. The author has observed that supply chain managers like numbers. Many managers want to convert the data from each animal welfare audit into a single score for each farm or slaughter plant. One way to successfully do this, is to have two different percentage levels for the most important animal-based measures. There would be a high automatic fail level and a lower level that would be a substantial number of points off. This could be done with measures for lameness, body condition score, animal cleanliness and other key welfare indicators, There are certain egregious abusive acts which should always result in an automatic failure. The number of points off for different key welfare indicators must be calculated so that a combined aggregate score does not conceal a serious welfare problem.

### 2.6. Emphasize Conditions an Auditor Can Directly Observe

In the author’s work as a welfare consultant to commercial supply managers, she recommends putting the most emphasis on conditions that can be directly observed. A farm either has the specified housing or does not have it. Animal-based key welfare indicators can be easily observed. There are many scoring tools available for different species. Some of the examples of readily available scoring tools are feather condition in laying hens [25], lameness in cattle [26,27], hock swelling in dairy cows [9,28] and gait score in broiler chickens [29]. In systems where the farms are integrated with a slaughter facility, many on-farm measurements can be easily done at the slaughter plant [30]. A common mistake in developing an effective commercial supply chain welfare auditing program, is an over reliance on data from records. The author has been a welfare auditor for many years and she has observed many falsified records. Unfortunately, falsified records are very common.

The OIE and other welfare assessments also put a lot of emphasis on having animal-based outcome measures [18] or measurables. For example, instead of specifying exactly how to design a dairy cow cubicle (free stall), the outcome of either poorly designed or poorly managed cubicles is assessed. Problems with cubicle design or management would result in a greater percentage of lame cows or cows with swollen hocks [28]. A recent easy-to-use dairy farm welfare assessment has been developed in the Netherlands [21]. It contains specifications for freestall dimensions. This resource measurement will work well in the Netherlands but it may not be effective in a worldwide supply system with many types of dairies. In these situations, a greater reliance on outcome measures may be required.

## 3. Incorporating the Five Domains into Existing Commercial Welfare Auditing Programs

For supply managers who are already using Welfare Quality assessments, the Five Domains will be easy to implement. As discussed in the Introduction, the first four domains are very similar to Welfare Quality [9]. Welfare Quality has four welfare areas of good feeding, good housing, good health, and appropriate behavior. The four domains listed above all influence the Fifth Affective Domain, which cannot be directly measured [1,2].

### 3.1. Animal-Based Outcome Measures for the Four Domains That Can Be Directly Assessed

#### 3.1.1. The First Domain Nutrition

Body condition scoring is one method for assessing good nutrition. For example, there are many different body condition scoring tools for assessing dairy cows [9,31,32,33]. All of these tools are slightly different, and a supply chain manager has to specify which specific tool is used in their system. For beef cows living on arid pastures, it is essential to assess body condition to insure they are eating sufficient feed. Body condition of breeding sows, ewes, and all farm animals needs to be assessed to insure that they are getting sufficient feed [34,35,36,37]. To help improve interobserver reliability, visual scoring charts should be made available to welfare auditors. These photographic visual aids will make it easy for auditors to identify noncompliant skinny animals. Auditors should be encouraged to have these charts on either their phone or laminated cards. They should refer to them often. Nutrition is not the only cause of poor body condition. Parasites or disease may also reduce body condition [38,39]. The Welfare Quality system puts a lot of emphasis on both clean water and animal cleanliness. Their protocols have good photographic tools for scoring water trough cleanliness [9]. 

#### 3.1.2. The Second Domain Environment

The second domain covers both the environment in a handling facility and the environment in housing. Problems with animal handling may be associated with deficiencies in the environment such as slick floors [40] or design mistakes in the handling facility [40]. Two important measurables that can be scored numerically during animal handling are slipping and falling [15,18,19,41,42]. Animals may become injured or stressed if they fall. Ease of animal movement through a handling facility, on a farm or at a slaughter plant can be assessed by counting the numbers of animals, turning back, stopping, balking or refusing to move forward [9,19,43]. 

If the housing is too hot, outcome measurables such as panting in cattle and sheep can be used as indicators of heat stress [19,44,45]. Cold stress in piglets can be assessed by observing huddling or piling [46]. Another important measure is cleanliness of the hide or feathers of both livestock and poultry. Animals that are lying in wet manure may have poor welfare and lack a positive affective state. Some examples of hygiene scoring tools can be found in [9,11]. In both cattle and poultry, a poor environment may also be associated with damage to either the skin or feathers. In broiler chickens, poor conditions of the litter can be assessed by measuring the percentage of birds with breast blisters [47] and foot pad lesions [48]. Groups of broilers with foot pad and lung lesions had more deads on arrival at the abattoir [49]. In laying hens, feather damage associated with housing can be assessed [25,50]. Another important variable which should be evaluated is atmospheric ammonia levels. High ammonia levels are detrimental to welfare [51]. 

In every supply chain there will be requirements and specifications for the types of housing that are permitted. Europe has already banned sow gestation stalls [52] but in other countries, they are still allowed. Many food companies have specifications that require group sow housing for gestating sows. In pork supply chains where group housing is required, there needs to be clear guidance on whether individual gestation stalls can be used for a short period of time for breeding and pregnancy conformation. Both the supply chain managers and the auditors need clear guidance on types of housing systems that are not allowed. It is important to avoid being too prescriptive on housing design because producers need to have the freedom to innovate. For laying hens, there are many types of systems that are now available to replace small battery cages [53]. When evaluating mortality data from different types of hen housing systems, the effect of a producer’s experience with a new system has to be taken into account. When a producer gains more experience, mortality often decreases [53].

In high animal welfare product lines, there also needs to be clear guidance on both bedding and environmental enrichment requirements. For example, the author has visited both excellent and really dirty straw bedded systems for pigs and cattle. In the dirty facilities, the producer did not use sufficient straw to keep the animals clean. Scoring animal cleanliness would have quickly detected this problem. Guidance is also required for environmental enrichment. It is beyond the scope of this paper to provide a review of all of the research on environmental enrichment. Below are some examples of typical environmental enrichments. For pigs, a variety of objects that they can chew are now commercially available [54]. Pigs prefer environmental enrichment devices that are chewable [54]. A ball on a chain is not effective [55]. The author has observed broiler chickens actively using ramps where they can either climb on top or hide under them. Another enrichment device that broilers will actually use are peck stones. For cattle or sheep, pasture access is required in many high welfare programs. One study showed that pasture may be a highly rewarding environment [56] Two of the most innovative enrichments are laser beams for broilers [57] and motorized grooming brushes for dairy cows. Research clearly shows that cows are highly motivated to use the brushes [58]. The motorized grooming brush would be an example of an animal experiencing a positive affective state [58].

#### 3.1.3. The Third Domain Health

Good health is essential to have good welfare, but health alone is not sufficient. An animal can be healthy and free of disease and still engage in abnormal repetitive stereotypic behavior [59]. On each farm, data must be collected on both mortality and morbidity for all species of animals. Lameness (difficulty walking) has been placed in the health domain because it may be associated with either disease [60] or deficiencies in the environment [28]. Many welfare specialists consider lameness to be one of the most serious welfare issues [61,62,63]. There are many scoring tools available that have been previously discussed. It is important for a supply chain manager to use the same scoring tool throughout their supply chain. Some tools use a “0” for the best rating and others use a “1” for best rating. Some of the popular lameness scoring tools for cattle are in [9,27,63,64]. Information on scoring lameness in sheep can be easily found [65]. Leg problems are really common in broiler chickens. Information on leg problems and lameness in broiler chickens can be found in [29]. 

Any condition that causes pain is included in the Health Domain [1,2], such as broken bones and bruises during handling and transport. Painful procedures such as dehorning and castration are also included in the Health Domain [1,2]. Animals can definitely experience and feel pain [66]. This would create a negative affective state in the Fifth Domain. Providing analgesics for pain relief definitely reduced indicators of stress such as high cortisol levels in the blood [66]. Research also showed that beak trimming in chickens may cause long-term pain [67]. To assess bruising, there are many scoring tools that are available [68]. The acts of abuse that were described earlier in this paper, would be included in the Health Domain because they cause pain. The effectiveness of stunning methods at slaughter and euthanasia on the farm would also be in the Health Domain. Poor stunning or poor euthanasia causes pain. There are many guidelines for assessing the effectiveness of stunning at slaughter [4,15,69]. Some of the problems that can be easily monitored at the slaughter plant are foot pad lesions in poultry, lameness in livestock and dirty animals [30,70]. Monitoring of compliance with housing requirements and the use of analgesics after surgery cannot be assessed at a slaughter plant. Most of the currently available scoring tools assess conditions that would cause pain or discomfort. There is a need to also have easy-to-use assessments for positive experiences.

#### 3.1.4. The Fourth Domain Behavioral Interactions

Mellor has split the Behavioral Interaction domain into three parts [1]. They are: (1) Animal Interactions with the Environment, (2) Animal Interactions with other Animals, and (3) Animal Interactions with People [2]. Some examples of outcome measurables for animal interactions with the environment may be either repetitive stereotypic behavior (negative) [71] or motivation to use enrichment devices (positive) [72]. Some of the most highly motivated behavioral needs are: nest boxes for laying hens [73] materials for pigs to chew or root [54], motorized brushes for cows [58] and devices for broilers to hide under, climb on, or peck [74]. Supply managers will need to rely on evidence from research studies, to determine which enrichments that farms in their supply chain should be required to have.

For the second category on interactions with other animals, there have been many studies on damaging behaviors inflicted by penmates or flockmates. Damage can result from feather pecking in hens [75], wounds from fighting in pigs [76] and tail biting in pigs [77]. A recent study showed that sows from one genetic line can be selected to be less aggressive and have fewer injuries when they are mixed [76]. Unfortunately, the piglets from the less aggressive genetic line had lower survivability [76]. Breeding to reduce aggression in group housed sows reduced piglet numbers. It is possible that this issue may not occur in all sow genetic lines. Breeders are challenged to think about difficult tradeoffs between animal welfare and economics. All of the above, would be examples of behaviors that would lead to a negative affective state. There is also need to assess positive behaviors between animals such as grooming each other [78].

For assessing the third section on interactions with humans, there are many handling scoring systems for assessing handling [9,15,69,79,80]. Numerical scoring of animal handling is already being used by many companies such as McDonalds [5] and commercial third party auditing companies. The following variables are scored in livestock handling systems that are used for loading trucks, vaccination or moving animals to the stunner at a slaughter plant. They are easy to measure indicators of poor handling practices that compromise animal welfare. Some examples of common measurements are the percentage of animals moved with electric prod (goad) [30,42,43,80], and vocalization during handling and restraint of cattle [30,43,79,81,82]. Vocalization during handling in cattle is associated with high cortisol levels [81,82]. Squealing in pigs is also used to assess handling practices [9,15]. In pigs, squealing is associated with physiological measures of stress [83]. Another measure that has been used in both cattle and sheep is speed of exiting after an animal is released from restraint [84,85,86,87]. High speeds are associated with greater stress. The percentage of animals turning back during handling or falling is also part of many assessments of animal handling [9,15,69]. These two variables have already been discussed in the Environment Section. Good stockmanship practices such as moving small groups of cattle and not yelling resulted in improved scores on handling measures [88]. 

All of the above handling measurements assess negative interactions between stock people and animals. There is also evidence that both positive attitudes by handlers and positive interactions between people and animals improved both productivity and welfare [89,90,91]. Dairy cows with a low somatic cell counts were more willing to approach people [28]. There is a need to develop simple measures for positive interaction between people and animals. Most of the measures which are currently being used to assess welfare in commercial supply chains are used to prevent suffering.

## 4. Summary of Key Welfare Indicators for Use with the Five Domains

Table 1, Table 2, Table 3, Table 4 and Table 5 contain key welfare indicators for cattle, swine, broiler chickens, and laying hens. References to both scientific studies and easy-to-use scoring tools are included on the tables. When welfare auditors have to be trained in a very short time, the use of videos and pictorial scoring aids is really useful. Recently one of the author’s former graduate students was hired as a welfare auditor. He is now traveling all over the U.S. and assessing slaughter plants, dairies, laying hens, broiler chickens, and pig farms. To help him evaluate body condition score, feather condition, lameness, and other key welfare indicators, the author helped him download some of the most useful online scoring tools. References for these scoring tools are in Table 1, Table 2, Table 3, Table 4 and Table 5. Information that is available on open access is given priority on the tables. Corporate managers often stop at paywalls. Their reading of scientific literature is often limited to open access materials. In many countries, managers cannot afford to pay fees to download papers. In the future, new welfare scoring tools will be developed. The author encourages open access publishing of scoring tools used to assess animal welfare.

In the future, many of the key welfare indicators will be able to be evaluated at slaughter with camera systems and artificial intelligence programs. These systems are already being developed for scoring foot pad lesions in broiler chickens [92,93]. In pigs, there are systems for assessing tail damage in pigs [94] and body condition in cattle [95]. 

## 5. The Importance of Commitment by Both Corporate Supply Chain Managers and Upper Management

During the last twenty-five years, the author has worked with both training animal welfare auditors and has served on the welfare panels of many large corporations, some of which include McDonald’s Corporation, Wendy International, Tyson, Costco Foods, Maple Leaf Foods and others. The most effective programs have managers who are committed to improving animal welfare. The author has observed either an improvement in a company’s program, or a decline with a change in top management. When the corporate welfare auditing programs started in the late 90s, the author took executives from McDonald’s and other companies on their first tours of farms and slaughter houses. During these initial tours, the author observed that animal welfare changed from being an abstract issue that was delegated to the legal or public relations department, to real issue that needed to be addressed. When one executive observed debilitated, emaciated dairy cows going into their product, they were appalled. Seeing bad animal welfare, motivated them to start auditing programs and implement improvements. A retired executive from McDonalds, has written a book about his experiences with starting the McDonald’s slaughter plant audits [112]. Recently I was with a top animal welfare executive from another company. We were visiting a beef slaughter plant that he purchased from. He became upset when he saw skinny emaciated organic cull dairy cows. This motivated him to make improvements.

To be effective, corporate buyers have to get out of the office and see what is occurring on farms and in slaughter houses. Reading third party auditor reports in the office is not sufficient. Over the years, the author has observed that the best programs have senior executives who actually visit their supplier’s facilities. The author has to disclose that she is currently a paid consultant for McDonald’s Corporation and Costco Corporation. For over twenty years, she has continued to observe a re-occurring pattern. Big corporations have constant shifts in top management. She has observed that the effectiveness of their animal welfare program may vary depending on the objectives of top managers. The greatest improvements occur when motivated supply chain managers are allowed to get out on the farms and make changes. Most supply chain managers and animal welfare officers the author has worked with want to make improvements. Recently the author talked to a supply chain person who felt that upper management held them back. That person was told to wait until upper management changed, and then they may have a window of opportunity to make significant improvements. Supply chain managers and animal welfare officers who work for large corporations are in a position to greatly improve animal welfare on farms and in slaughter houses. The information in this paper will help them to implement effective programs.

## 6. Conclusions

The Five Domains Framework can be easily incorporated into many existing animal welfare programs conducted by commercial supply chain managers. They can also be incorporated into programs of continuing improvement. Many supply chain managers can relate to The Five Domains because it goes beyond the prevention of suffering.

## Figures and Tables

**Table 1:** Guide to Assessment Tools for Key Welfare Indicators for Beef Cattle.

Domain	Parameter Assessed	References
First Nutrition	Body Condition—Breeding cows	[96]
	Water trough cleanliness	[97]
Second Environment	Slipping and falling during handling	[15,18,98]
	Turning back balking during handling	[97]
	Heat stress—Open mouth breathing	[44,45]
	Hygiene Scoring—Dirty hide	[97]
	Housing Requirements and Specifications	
Third Health	Lameness—All types of cattle	[20,26,64,99]
	Bruise scoring	[68]
	Effectiveness of stunning at slaughter	[15,69]
	Swollen hocks	[97]
Fourth Behavior	Electric prod use during handling	[15,69,79,98]
	Vocalization during handling	[15,69,79,98]
	Acts of abuse	[15,16,17,18]
	Animal refusing to move forward during handling	[43,97]

**Table 2:** Key Welfare Indicators for Dairy Cows.

Domain	Parameter Assessed	References
First Nutrition	Body condition of dairy cows	[21,33,100,101]
	Water trough cleanliness	[21,97]
Second Environment	Slipping and falling during handling	[15,16,17,18]
	Hygiene scoring—Dirty hide	[21,100]
	Swollen hocks	[21,28,102]
	Housing Requirements and Specifications	
Third Health	Lameness	[21,27,103]
	Bruises at slaughter	[68]
	Effectiveness of stunning at slaughter	[15,69]
Fourth Behavior	Electric prod use during handling	[15,69,79,98]
	Vocalization during handling	[15,69,79,98]
	Flight distance from people	[21]
	Acts of abuse	[15,16,17,18]
	Environmental enrichment requirements	[58]

**Table 3:** Key Welfare Indicators for Pigs.

Domain	Parameter Assessed	References
First Nutrition	Body condition of breeding sows	[34]
	Water trough cleanliness	[104]
Second Environment	Slipping and falling during handling	[15,18]
	Shoulder lesions	[105]
	Swollen joints and hoof damage	[106]
	Huddling—Cold stress piglets	[10,105]
	Housing requirements and specifications	
Third Health	Lameness sows and finishing pigs	[106,107]
	Skin damage	[70,108]
	Effectiveness of stunning at slaughter	[15,69]
	Hernias	[104] [70,77]
	Tail damage	
	Housing requirements and specifications	
Fourth Behavior	Electric prod using during handling	[15]
	Vocalization scoring during handling	[15]
	Lesions from fighting	[105]
	Stereotypic behavior	[59]
	Acts of abuse	[15,16,17,18]
	Environmental enrichment	[54,55]

**Table 4:** Key Welfare Indicators for Laying Hens.

Domain	Parameter Assessed	References
First Nutrition	Water	
Second Environment	Feather condition scoring	[25]
	Foot pad lesions	[109]
	Wounds	[25]
	Foot pads	[25]
	Housing requirements and specifications	
Third Health	Beak abnormalities Wounds	[109] [25]
Fourth Behavior	Feather pecking	[25]

**Table 5:** Key Welfare Indicators for Broiler Chickens.

Domain	Parameter Assessed	References
First Nutrition	Water	[109]
Second Environment	Breast blisters	[110]
	Hock burn	[110]
	Foot pad lesions	[48,110]
	Cleanliness of plumage	[47,110]
	Space—Day of catch – Birds can move away 1 m	
	Housing requirements and specifications	
Third Health	3 point gait scoring	[29,61,109,111]
	Bruise scoring	
	Effectiveness of stunning	
Fourth Behavior	Environmental enrichments	[57]
	Acts of abuse	
	Broken wings due to poor handling	[11]
